# Impact of Intermittent Preventive Treatment With Dihydroartemisinin-Piperaquine on Malaria in Ugandan Schoolchildren: A Randomized, Placebo-Controlled Trial

**DOI:** 10.1093/cid/ciu150

**Published:** 2014-03-12

**Authors:** Joaniter I. Nankabirwa, Bonnie Wandera, Pauline Amuge, Noah Kiwanuka, Grant Dorsey, Philip J. Rosenthal, Simon J. Brooker, Sarah G. Staedke, Moses R. Kamya

**Affiliations:** 1 Department of Medicine, Makerere University College of Health Sciences; 2 Infectious Disease Research Collaboration; 3 Department of Epidemiology and Biostatistics, School of Public Health, Makerere University College of Health Sciences, Kampala, Uganda; 4 Department of Medicine, San Francisco General Hospital, University of California, San Francisco; 5 Faculty of Infectious and Tropical Diseases, London School of Hygiene and Tropical Medicine, United Kingdom; 6 Malaria Public Health and Epidemiology Group, Kenya Medical Research Institute–Wellcome Trust Collaborative Programme, Nairobi, Kenya

**Keywords:** malaria, intermittent preventive treatment, schoolchildren

## Abstract

***Background.*** Intermittent preventive treatment (IPT) in schoolchildren offers a promising option for malaria control. However, the optimal drug and dosing regimens for IPT remain to be determined.

***Methods.*** We conducted a randomized, double-blind, placebo-controlled trial in 740 schoolchildren aged 6–14 years living in a setting of high malaria transmission in Uganda. Enrolled children were randomized to dihydroartemisinin-piperaquine (DP) given once a month (IPTm), DP given once a school term (4 treatments over 12 months, IPTst), or placebo and followed for 12 months. The primary outcome was the incidence of malaria over 12 months. Secondary outcomes included parasite prevalence and anemia over 12 months. Analyses were conducted on an intention-to-treat basis.

***Results.*** In the placebo arm, the incidence of malaria was 0.34 episodes per person-year and the prevalence of parasitemia and anemia was 38% and 20%, respectively. IPTm reduced the incidence of malaria by 96% (95% confidence interval [CI], 88%–99%, *P* < .0001), the prevalence of asymptomatic parasitemia by 94% (95% CI, 92%–96%, *P* < .0001), and the prevalence of anemia by 40% (95% CI, 19%–56%, *P* < .0001). IPTst had no significant effect on the incidence of symptomatic malaria or the prevalence of anemia, but reduced the prevalence of asymptomatic parasitemia by 54% (95% CI, 47%–60%, *P* < .0001).

***Conclusions.*** Monthly IPT with DP offered remarkable protection against clinical malaria, parasitemia, and anemia in schoolchildren living in a high-malaria-transmission setting.

***Clinical Trials Registration.***  NCT01231880.

## Abstract (precis)

Dihydroartemisinin-piperaquine administered at monthly intervals, but not that dosed once a school term, is a remarkably effective measure for the prevention of incidence of malaria, prevalence of parasitemia, and prevalence of anemia in schoolchildren living in a high-transmission setting.

Children of school age (5–14 years) benefit relatively little from current malaria control interventions, due to a focus on younger children and pregnant women [1, 2]. Yet, in Africa, 20%–50% of school-aged children experience malarial episodes each year [3], with a negative impact on school attendance and educational performance [4, 5]. In high-transmission settings, up to 70% of school-aged children harbor malaria parasites, causing anemia [6], cognitive impairment [7, 8], and maintenance of reservoirs that promote transmission to others. Clearly, malaria control would be a valuable addition to school health programs.

One intervention under investigation is intermittent preventive treatment (IPT), the administration of curative doses of antimalarials at predefined intervals regardless of infection status. IPT targeted at schoolchildren has resulted in reduced rates of malaria, asymptomatic parasitemia, and anemia, as well as improved cognitive function [9–13]. However, the optimal drug and dosing regimen are uncertain. We previously showed that, in a region of high malaria transmission, a single 3-day course of dihydroartemisinin-piperaquine (DP) better prevented malarial parasitemia over 42 days and was better tolerated than either sulfadoxine-pyrimethamine (SP) or amodiaquine plus sulfadoxine-pyrimethamine (AQ + SP) [14]. We herein report results of a trial evaluating DP, either monthly (IPTm) or once per school term (IPTst), in schoolchildren in a high-malaria-transmission setting in Uganda.

## METHODS

### Study Design, Setting, and Population

This randomized, double-blind, placebo-controlled trial was conducted between February 2011 and February 2012 in Mulanda Primary School, Tororo District, in eastern Uganda. Tororo District is characterized by high-intensity year-round malaria transmission, principally of *Plasmodium falciparum*, with an estimated entomological inoculation rate of 562 infective bites per person per year [15]. In the 5 years prior to this study, malaria control in Tororo District was typically limited to the promotion of IPT during pregnancy, distribution of insecticide-treated nets (ITNs) through antenatal care services, and malaria case management with artemisinin-based combination therapy. In January 2011, a community-based mass campaign to distribute free ITNs was conducted.

Mulanda subcounty has 8 primary schools. Mulanda Primary School was selected due to its large population (1320 students) and close proximity (approximately 500 m) to the main public health facility, Mulanda Health Centre IV. Meetings were held to explain the nature and purpose of the trial to teachers and parents or guardians, and written informed consent was obtained. All children with consent from their parent/guardian were screened for eligibility. Children were excluded if they had (*i*) known allergy or adverse reaction to artemisinin-based regimens; (*ii*) history of menarche; (*iii*) fever (axillary temperature ≥37.5^°^C) or history of fever in the previous 24 hours; (*iv*) evidence of severe malaria or danger signs; or (*v*) ongoing antimalarial treatment. Recruitment was done once and closed after baseline assessments.

### Enrollment Procedures

At enrollment, a standardized questionnaire was administered to children fulfilling selection criteria to obtain data on sociodemographics and bed-net ownership and use. A standardized assessment of symptoms and a focused physical examination including measurement of weight and temperature were conducted. A finger-prick blood sample was obtained to assess for *Plasmodium* infection by thick and thin blood smear and for hemoglobin estimation. A stool sample was collected to assess for helminth infection. All children received a long-lasting ITN, and a single dose of albendazole (400 mg) was administered.

### Study Drugs and Treatment Allocation

A randomization list was computer-generated using fixed blocks of 12 by an individual not involved in patient care. Participants were randomized using previously prepared, consecutively numbered, opaque sealed envelopes and assigned study numbers sequentially. A nurse not involved in care allocated the study group after opening the correspondingly numbered envelope. All other study personnel were blinded to study group assignments, and children were not informed of their regimen, but the color and taste of study medication and placebo were dissimilar. Study drugs were dosed as DP (Duo-Cotecxin, Holley-Cotec Pharmaceuticals, Beijing, China) once daily for 3 consecutive days each month (IPTm), or at the beginning of each school term (February 2011, May 2011, August 2011, and January 2012 [IPTst]). DP was dosed using weight-based guidelines targeting a total dose of 6.4 mg/kg dihydroartemisinin and 51.2 mg/kg piperaquine, given with glucose biscuits. Placebo was administered to the placebo and IPTst groups to simulate the IPTm dosing schedule. All treatment was administered at school by the study nurses and was directly observed. After drug administration, children were observed for 30 minutes, and treatment was readministered if vomiting occurred.

### Follow-up Visits

Finger-prick blood smears were performed monthly to assess for *Plasmodium* infection. At the start of each school term, standardized history and physical examination and assessments for hemoglobin were performed prior to treatment. Children absent for any scheduled visits were followed up at home. School attendance was assessed daily by roll call by teachers, with absent children followed up at home by study personnel to establish reasons for absenteeism. Children who were absent due to illness were taken to the study clinic for diagnosis, management, and documentation. Adverse events were assessed and graded according to standardized criteria. Children with illnesses other than malaria received standard of care or were referred to Mulanda Health Centre. Medications with antimalarial activity were avoided whenever possible.

### Recording of Clinical Malaria Episodes

Monitoring for malaria was by active case detection. Sick children were identified by teachers and sent to the study clinic and absent children were visited at home and taken to the study clinic if ill. The clinic was open 7 days a week. Children with documented fever (axillary temperature ≥37.5°C) or history of fever in the previous 24 hours had a blood sample for thick and thin smears. Malaria was defined as fever (documented or by history) with any quantity of malaria parasites on the blood smear. Episodes of uncomplicated malaria were treated with artemether-lumefantrine, the recommended first-line treatment in Uganda, following standard dosing guidelines and under supervision of the study nurse. Treatment failures within 14 days of prior therapy were treated with oral quinine 10 mg/kg every 8 hours for 7 days. Episodes of complicated malaria (severe malaria or danger signs) were referred to Mulanda Health Centre IV for intravenous quinine.

### Laboratory Evaluations

Blood smears were labeled, air dried, and stained with 2% Giemsa for 30 minutes. Parasite densities were determined from thick blood smears by counting asexual parasites per 200 white blood cells (or per 500 white blood cells if the count was <10 parasites per 200 cells), assuming a white blood cell count of 8000 cells/mL. A smear was considered negative if no parasites were seen in 100 high-powered fields. Gametocytemia was determined using the same methodology. Thin smears were viewed for species identification. Two independent and experienced microscopists read the slides, with a third microscopist resolving discrepancies. Stool examination was by the Kato-Katz technique to assess for helminth infections. Hemoglobin concentration was assessed using a portable hemoglobinometer (HemoCue Ltd, Angelholm, Sweden) and estimated to 0.1 g/dL. Follow-up for malaria, anemia, and parasitemia were conducted for 12 months after enrollment. Participants were withdrawn for change of school, withdrawal of informed consent, or noncompliance with study procedures.

### Statistical Analysis

The study was designed to test the hypothesis that IPTm would reduce the incidence of malaria compared with placebo by 33% or greater. We assumed that the incidence of malaria would be 0.57 episodes per person year in the placebo arm [14], and thus we would need to enroll 228 participants in each arm to detect our targeted protective efficacy with 80% power at 95% significance, allowing for 10% loss to follow-up.

Data were double-entered and cross-checked in a bespoke Microsoft Access database (Microsoft Corp, Seattle, Washington). Consistency checks were performed and all discrepancies and queries verified against original paper forms. Statistical analyses were done using Stata version 12.0 software (StataCorp, College Station, Texas). All analyses used an intention-to-treat approach. Incidence outcomes were compared using a negative binomial regression model, and prevalence outcomes were compared using generalized estimating equations with adjustment for repeated measures in the same study participant. Measures of association were expressed as incidence rate ratios, prevalence ratios, and mean differences. A *P* value ≤.05 was considered statistically significant. The primary outcome was the incidence of malaria, defined as the number of incident episodes per time at risk. Time at risk was calculated from the day following the initiation of study drugs to the last day of observation, minus 14 days after each episode of malaria, a standard approach. Malaria within 1 month of IPTst was defined as any episode in March, June, or September, within 2 months as any episode in April, July, or October, within 3 months as any episode in May, August, or November, and within 4 months as any episode in December.

Secondary outcomes included prevalence of parasitemia, defined as the number of positive smears divided by the total number of smears over 1 year. Parasitemia after IPTst was defined as described above for malaria. Anemia was defined as <11.5 g/dL for children aged 6–11 years and <12.0 g/dL for those aged 12–14 years [16].

An adverse event was defined as any untoward medical occurrence, irrespective of its suspected relationship to study medications. All events were graded by severity and relationship to study treatment [17]. The risk of experiencing an adverse event was estimated, and comparisons with placebo were made using χ^2^ tests.

### Role of the Funding Source and Ethical Approval

The sponsors had no role in study design, data collection, data analysis, data interpretation, or writing the report. The corresponding author had full access to study data and final responsibility for submission of the manuscript. Ethical approval was obtained from the Uganda National Council for Science and Technology and the Makerere University School of Medicine Research and Ethics Committee.

## RESULTS

### Study Subjects and Follow-up

**Figure 1.**
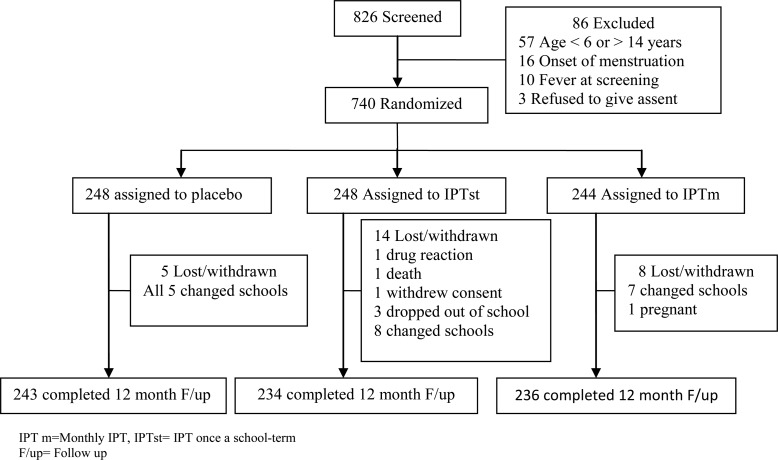
Trial profile. Abbreviations: F/up, follow-up; IPTm, intermittent preventive treatment once a month; IPTst, intermittent preventive treatment once a school term.

Between January and February 2011, 826 children were screened for eligibility, and 740 (89.6%) met inclusion criteria and were randomized to study arms (Figure 1). Baseline characteristics of participants were similar across the study arms (Table 1). A total of 713 (96.4%) children completed 1 year of follow-up. Among the children lost during follow-up, 20 changed schools, 3 withdrew (1 hypersensitivity reaction after the first course of treatment, 1 pregnancy, and 1 withdrawal of consent), 3 dropped out of school, and 1 died (Figure 1).

**Table 1.** Baseline Characteristics of the Study Population by Treatment Group

Characteristic	Study Group		
	Placebo	IPTst	IPTm
No. of children	248	248	244
Sex, female, No. (%)	129 (52.0)	139 (56.1)	126 (51.6)
Age at enrollment, y, mean (SD)	9.8 (2.3)	9.7 (2.2)	9.9 (2.1)
Weight, kg, mean (SD)	28.6 (8.4)	28.4 (8.2)	28.6 (8.2)
Hemoglobin, g/dL, mean (SD)	11.7 (1.0)	11.7 (1.1)	11.6 (1.0)
Household owning at least 1 bed-net, No. (%)	224 (90.3)	218 (87.9)	220 (90.2)
Bed-net use in last 24 h, No. (%)	91 (36.7)	87 (35.1)	84 (34.4)
Asymptomatic parasitemia			
All *Plasmodium* species, No. (%)	79 (31.9)	73 (29.4)	71 (29.1)
*P. falciparum*	70	70	64
*P. malariae*	5	1	5
*P. vivax*	2	0	2
*P. ovale*	2	2	0
Geometric mean parasite density per µL	549	647	611
Gametocyte presence, No. (%)	9 (3.6)	6 (2.4)	7 (2.9)
Helminths present, No. (%)	21 (8.5)	13 (5.2)	22 (9.0)
Socioeconomic group, quartiles, No., (%)			
Poorest	67 (27.0)	62 (25.0)	67 (27.5)
Poor	85 (34.3)	82 (33.1)	74 (30.3)
Less poor	41 (16.5)	36 (14.5)	43 (17.6)
Least poor	55 (22.2)	68 (27.4)	60 (24.6)

Abbreviations: IPTm, intermittent preventive treatment once a month; IPTst, intermittent preventive treatment once a school term; SD, standard deviation.

### Efficacy Outcomes Following IPT

**Figure 2.**
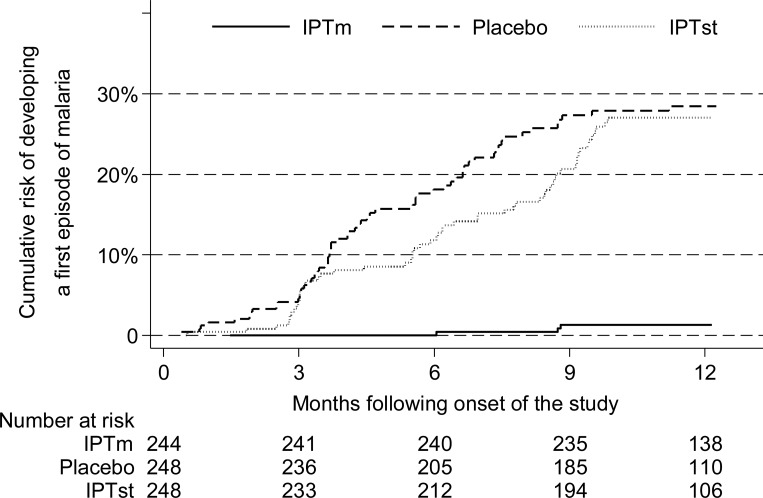
Cumulative risk of malaria over 1 year of follow-up by treatment group. Abbreviations: IPTm, intermittent preventive treatment once a month; IPTst, intermittent preventive treatment once a school term.

There were 167 incident episodes of malaria over 12 months of follow-up (Table 2). The incidence of malaria was significantly lower in the IPTm arm compared with the placebo arm (0.01 vs 0.34 episodes per person per year, respectively), resulting in a 96% protective efficacy (incidence rate ratio [IRR], 0.04; 95% confidence interval [CI], .01–.12; *P* < .0001). Compared with the placebo arm, the overall incidence of malaria (Table 2) or the cumulative risk of developing at least 1 episode of malaria (Figure 2) over 1 year of follow-up was not significantly different in the IPTst arm. However, compared with the placebo arm, the incidence of malaria was significantly lower within the first and second months after IPTst administration, resulting in 66% (IRR, 0.34; 95% CI, .19–.59; *P* < .0001) and 45% (IRR, 0.55; 95% CI, .30–.99; *P* = .037) protective efficacies within 1 and 2 months of IPTst administration, respectively. Restricting the case definition of malaria to fever and >2000 parasites/µL of blood yielded similar results and comparisons (Table 2).

**Table 2.** Clinical, Parasitological, and Hematological Outcomes by Treatment Group After 1 Year of Follow-up

Outcome	Study Group										
	Placebo			IPTst				IPTm			
	Events	PYAR	Incidence	Events	PYAR	Incidence	IRR IPTst vs Placebo (95% CI)	Events	PYAR	Incidence	IRR IPTm vs Placebo (95% CI)
Clinical malaria											
All episodes	83	242.7	0.34	81	238.1	0.34	1.00 (.68–1.47)	3	240.5	0.01	0.04 (.01–.12)
Parasite density >2000/µL	49	242.7	0.20	63	238.1	0.26	1.32 (.82–2.14)	1	240.5	0.004	0.02 (.003–.15)
Episodes within 1 mo of IPTst^a^	38	94.8	0.40	12	93.5	0.13	0.31 (.15–.63)	0	94.3	0.00	0
Episodes within 2 mo of IPTst^a^	22	56.5	0.39	14	56.5	0.25	0.62 (.29–1.32)	0	56.4	0.00	0
Episodes within 3 mo of IPTst^a^	21	57.1	0.37	38	55.4	0.69	1.96 (1.06–3.62)	2	56.4	0.04	0.09 (.02–.42)
Episodes within 4 mo of IPTst^a^	2	34.2	0.06	17	32.8	0.52	9.37 (2.09–42.1)	1	33.5	0.03	0.51 (.05–5.72)
Outcome	Events	Total Tests	Prevalence	Events	Total Tests	Prevalence	Prevalence Ratio IPTst vs Placebo (95% CI)	Events	Total Tests	Prevalence	Prevalence Ratio IPTm vs Placebo (95% CI)
Parasitemia											
All events	1037	2700	0.38	472	2644	0.18	0.46 (.40–.53)	58	2638	0.02	0.06 (.04–.08)
Events within 1 mo of IPTst^a^	326	981	0.33	23	960	0.02	0.07 (.05–.11)	19	960	0.02	0.06 (.04–.10)
Events within 2 mo of IPTst^a^	297	738	0.40	126	726	0.17	0.43 (.35–.53)	14	721	0.02	0.05 (.03–.09)
Events within 3 mo of IPTst^a^	307	737	0.42	238	720	0.33	0.79 (.69–.92)	20	719	0.03	0.06 (.04–.11)
Events within 4 mo of IPTst^a^	107	244	0.44	85	238	0.36	0.81 (.65–1.02)	5	238	0.02	0.05 (.02–.12)
Anemia											
All events	148	736	0.20	125	716	0.17	0.86 (.66–1.13)	87	717	0.12	0.60 (.44–.81)
Gametocytemia											
All events	129	2700	0.05	54	2644	0.02	0.43 (.31–.58)	11	2638	0.004	0.09 (.05–.16)

Abbreviations: CI, confidence interval; IPTm, intermittent preventive treatment once a month; IPTst, intermittent preventive treatment once a school term; IRR, incidence rate ratio; PYAR, person year at risk.

^a^ IPTst was administered in February, May, and August 2011 as well as January 2012. Events within 1 month of IPTst occurred in March, June, and September 2011. Events within 2 months of IPTst occurred in April, July, and October 2011. Events within 3 months of IPTst occurred in May, August, and November 2011. Events within 4 months of IPTst occurred in December 2011.

The prevalence of parasitemia, which was assessed monthly, was significantly lower in the IPTm (2%) and IPTst (18%) arms compared with the placebo (38%) arm, resulting in a 94% protective efficacy with IPTm (prevalence ratio [PR], 0.06; 95% CI, .40–.08; *P* < .001) and 54% protective efficacy with IPTst (PR, 0.46; 95% CI, .40–.53; *P* < .001) over 1 year of follow-up. Similar to what was observed with malarial incidence, the prevalence of parasitemia in the IPTst group increased with increasing duration after treatment. The prevalence of gametocytemia was significantly lower in the IPTm and IPTst groups compared with the placebo group (Table 2).

Mean hemoglobin increased between baseline and 1 year in all children. The mean increase in hemoglobin in the IPTst group did not differ from that in the placebo group (0.86 g/dL [interquartile range {IQR}, −0.1 to 1.8] vs 0.79 g/dL [IQR, −0.1 to 1.7]; *P* = .638). In contrast, the mean increase in hemoglobin in children in the IPTm group was significantly greater than that in the placebo group (1.20 g/dL [IQR, 0.1–2.15] vs 0.79 g/dL IQR −0.1 to 1.7; *P* = .003). The overall prevalence of anemia was significantly lower in the IPTm arm (0.12) but not the IPTst arm (0.17), compared with the placebo arm (0.20), resulting in a 40% protective efficacy for IPTm (PR, 0.60; 95% CI, .44–.81; *P* = .030) (Table 2). Only 2 children had hemoglobin levels <8 g/dL during the 1-year follow-up period. One child, diagnosed with acute lymphoblastic leukemia, had severe anemia (hemoglobin level, 3.4 g/dL).

### Safety Outcomes

There was 1 death, due to acute lymphoblastic leukemia, in a 7-year-old randomized to IPTst. Fourteen serious adverse events were reported, 6 in the IPTm group, 5 in the IPTst group, and 3 in the placebo group. All serious adverse events were judged unlikely to be associated with treatment; the most commonly reported events were fractures. Mild events, such as fever, headache, nausea, vomiting, and diarrhea, which were commonly associated with malaria, were more frequent in the placebo group than the intervention arms (Table 3).

**Table 3.** Risk of Adverse Events in All Participants Over 1 Year of Follow-up by Treatment Group, and Pairwise Comparisons With Placebo

Event Description	Study Group				
	Placebo (n = 248)	IPTst (n = 248)		IPTm (n = 244)	
	% Risk (95% CI)	% Risk (95% CI)	*P* Value^a^	% Risk (95% CI)	*P* Value^a^
Any adverse event	92.7 (88.8–95.4)	90.7 (86.5–93.7)	.420	88.9 (84.4–92.3)	.145
AEs commonly related to DP					
Anorexia	1.2 (.4–3.5)	1.2 (.4–3.5)	1.000	1.6 (.6–4.1)	.706
Nausea	5.7 (3.4–9.3)	3.2 (1.6–6.2)	.193	0.8 (.2–2.9)	.003
Vomiting	11.3 (7.9–15.8)	9.7 (6.6–14.0)	.561	3.7 (2.0–6.9)	.001
Diarrhea	15.7 (11.7–20.7)	6.9 (4.3–10.7)	.006	8.2 (5.4–12.3)	.028
Pruritus	0.8 (.2–2.9)	1.2 (.4–3.5)	.654	0.8 (.2–2.9)	1.000
Commonest AEs reported					
Cough	72.9 (67.1–78.1)	66.5 (60.5–72.1)	.121	70.1 (64.1–75.5)	.491
Headache	55.7 (49.4–61.7)	50.0 (43.8–56.2)	.212	38.5 (32.6–44.8)	<.001
Fever	44.7 (38.7–50.9)	38.3 (32.5–44.5)	.205	25.0 (19.9–30.8)	<.001
Abdominal pain	29.8 (24.5–35.8)	29.0 (23.7–34.9)	.845	22.5 (17.8–28.2)	.066
Flu	33.9 (28.3–39.9)	29.0 (23.7–34.9)	.240	39.8 (33.8–46.0)	.175
Wounds	18.6 (14.2–23.9)	18.6 (14.2–23.8)	.977	17.6 (13.4–22.9)	.795
Skin rash	10.9 (7.6–15.4)	12.5 (8.9–17.2)	.579	11.1 (7.7–15.6)	.943
Joint aches	9.3 (6.3–13.5)	10.1 (6.9–14.5)	.763	7.8 (5.0–11.8)	.552
Sore throat	10.9 (7.6–15.4)	8.9 (5.9–13.1)	.456	8.6 (5.7–12.8)	.390
Weakness	8.8 (5.3–12.1)	4.8 (2.8–8.3)	.077	4.9 (2.8–8.4)	.087
Serious adverse events					
All SAEs	1.2 (.4–3.5)	2.0 (.8–4.6)	.478	2.5 (1.1–5.3)	.284
Fractures	0.4 (.07–2.3)	1.2 (.4–3.5)	.317	2.5 (1.1–5.3)	.051
Death	0	0.4 (.07–2.3)	.319	0	
Pneumonia	0.8 (.2–2.9)	0.4 (.07–2.3)	.564	0	.158

Abbreviations: AE, adverse event; CI, confidence interval; DP, dihydroartemisinin-piperaquine; IPTm, intermittent preventive treatment once a month; IPTst, intermittent preventive treatment once a school term; SAE, severe adverse event.

^a^ Reference group is placebo.

## DISCUSSION

School-aged children suffer consequences of malaria but are generally neglected in malaria control strategies. One control strategy under investigation for schoolchildren is IPT. We previously showed that, in Ugandan schoolchildren, a single course of DP was more efficacious than either SP or AQ + SP in preventing parasitemia over the subsequent 6 weeks [14]. In this study, we compared 2 DP dosing regimens and placebo in the same high-malaria-transmission setting. IPTm was highly efficacious, reducing the incidence of malaria by 96%, the prevalence of parasitemia by 94%, and the prevalence of anemia by 40%. IPTst only provided protection against malaria within the first 2 months after drug administration and against parasitemia within the first 3 months after drug administration. Our results suggest that DP administered at monthly intervals, but not dosed once a term, is a remarkably effective measure for the prevention of malaria in schoolchildren living in a high-transmission setting.

For IPT, a drug should eliminate circulating parasites and persist at sufficient levels to prevent multiplication of parasites acquired between doses. In our study, IPTm was highly efficacious in reducing parasitemia and preventing malaria. These findings are consistent with the long half-life of piperaquine [18]. Previous studies have shown that DP is highly efficacious for the treatment of malaria, with substantial posttreatment prophylactic efficacy in Africa [19, 20] and Asia [21]. In one study that considered less-frequent preventive dosing, monthly DP was more efficacious than a bimonthly regimen in preventing malaria in adults in Thailand [22]. Similarly, in our study, IPTm was more efficacious than IPTst. Taken together, available results suggest that a monthly dosing interval is most appropriate for IPT with DP [18, 22].

IPTm significantly improved hemoglobin levels and reduced the risk of anemia, probably due to effects against malaria. IPTst had no effect on anemia, in contrast to a previous study in Kenya, where the protective efficacy of IPTst with AQ + SP against anemia was 48% [9]. In the Kenyan study, parasite prevalence was 4.6% in children receiving IPTst, compared with 17.8% in our study. The explanation for differential impacts on parasitemia and anemia in the 2 studies may be differences in transmission intensities, the drugs used for IPT, or the socioeconomic status of study participants.

IPT with SP is recommended in pregnancy (IPTp) and in infants (IPTi) living in areas with moderate to high malaria transmission, whereas seasonal malaria chemoprevention (SMC) with monthly AQ + SP is recommended in children aged <5 years living in areas with highly seasonal malaria transmission in the Sahel subregion of Africa [23]. Although studies have demonstrated benefits of IPT and SMC with SP-containing regimens [9, 24, 25], widespread resistance to SP in Africa [26] may render these malaria control measures ineffective [14, 27]. DP may serve as a suitable alternative, especially for SMC, for which issues of tolerability to AQ + SP have also been raised [14]. However, before implementation, the efficacy, tolerability, and safety of DP for IPTp, IPTi, and SMC should be further evaluated.

Our results suggest that monthly administration of DP will have major benefits for schoolchildren living in areas with high malaria endemicity. However, some concerns regarding this approach need to be addressed. First, the feasibility of monthly administration of a 3-day treatment regimen may be questioned, but it is noteworthy that schools offer relatively simple settings for administration of directly observed therapies. Second, prevention of malaria through chemoprevention may impede the development of immunity, leading to rebound morbidity after completion of the intervention, although rebound after chemoprevention has generally either not been seen, or been modest [28], and it would likely be of less importance in schoolchildren than younger children. Third, chemoprevention with DP may select for parasites resistant to the components of the regimen, particularly piperaquine. Older studies reported resistance to piperaquine in China following years of heavy use [29]. Analysis of samples from a cohort study in Uganda [30], but not studies in Burkina Faso [31], showed selection after treatment with DP toward parasites with *pfmdr1* mutations associated with decreased sensitivity to other aminoquinolines. The impact of chemoprevention with DP on the selection of drug-resistant parasites is unknown. Surveillance of parasites emerging under DP-selective pressure is indicated, and analysis of samples from our trial is planned. Fourth, these findings may not be generalizable to medium- and low-transmission settings. Fifth, the lack of similarity between the placebo and DP may have unblinded study participants, but our outcome measures included clinical and laboratory evaluations by study personnel blinded to the participants' study arms. Considering our results in light of these concerns, further study of the feasibility, safety, and cost-effectiveness of IPT targeted at schoolchildren with DP in different transmission settings will be warranted, but nonetheless, implementation of this highly efficacious intervention in the near future is worthy of strong consideration.
